# Non-ST elevation myocardial infarction (NSTEMI) in three hospital settings in South Africa: does geography influence management and outcome? A retrospective cohort study

**DOI:** 10.5830/CVJA-2013-017

**Published:** 2013-06

**Authors:** Jane Moses, Anton F Doubell, Philip G Herbst, Hellmuth SVH Weich, Karl JC Klusmann

**Affiliations:** Division of Cardiology, Department of Medicine, Stellenbosch University and Tygerberg Hospital, South Africa; Division of Cardiology, Department of Medicine, Stellenbosch University and Tygerberg Hospital, South Africa; Division of Cardiology, Department of Medicine, Stellenbosch University and Tygerberg Hospital, South Africa; Division of Cardiology, Department of Medicine, Stellenbosch University and Tygerberg Hospital, South Africa; Department of Medicine, Stellenbosch University and Worcester Hospital, South Africa

**Keywords:** acute coronary syndrome, NSTEMI, myocardial infarction

## Abstract

**Background:**

Guidelines advise early angiography in non-ST elevation myocardial infarction (NSTEMI) to ensure an optimal outcome. Resource limitations in secondary hospitals in the Western Cape dictate a local guideline to treat NSTEMIs medically with out-patient assessment for angiography, unless mandatory indications for early angiography occur.

**Methods:**

A retrospective cohort study assessed NSTEMIs at Tygerberg Hospital (TBH), Karl Bremer Hospital (KBH) and Worcester Hospital (WH) over one year. Two cohorts were analysed, secondary hospitals (KBH and WH; SH) and secondary service within a tertiary hospital (TBH). Where differences were found, sub-analysis compared WH and KBH.

**Results:**

TBH and SH were similar at baseline and in clinical presentation. Cases at TBH were more likely to receive in-patient angiography (94 vs 51%, *p* < 0.0001), and had a lower in-patient mortality rate (6 vs 23%, *p* = 0.0326). There was no difference between KBH and WH in sub-analysis.

**Conclusion:**

This study confirmed that the management and mortality of NSTEMIs in the public health sector in the Western Cape, South Africa is not influenced by geography, but rather by the level of service available in the hospital of first presentation.

## Abstract

The European Society of Cardiology (ESC) guidelines state that patients presenting with an acute coronary syndrome (ACS) with raised cardiac markers and without ST-segment elevation (non-ST elevation myocardial infarction – NSTEMI), should receive early coronary angiography and revascularisation,^1^ as trials have shown clear mortality benefit for such an early invasive approach.^2^-^6^ The South African Heart Association is an affiliated member of the European Society of Cardiology (ESC) and therefore subscribes to its guidelines, but strict adherence is not always possible due to limited facilities and personnel.

The South African public health service is divided into three levels of care; primary care (managed by family physicians), secondary care (with certain specialists such as specialist physicians but without sub-specialist care), and tertiary care (provided by academic referral hospitals and with access to sub-specialist services such as cardiologists). These tertiary centres are usually located in large cities, resulting in inequality in the distribution of sub-specialist care. This may be detrimental to many patients presenting to secondary hospitals but the extent of this is unknown. Furthermore, the studies on which these guidelines are based were performed in the first world and may not be applicable to our patients or practice, even to those presenting primarily to sub-specialist centres.^7^-^10^

Current best-practice guidelines as practiced in secondary hospitals in the Western Cape suggest patients with NSTEMIs be admitted for medical management, including bed rest, antiplatelet treatment with aspirin, β-blockade, anti-coagulation with heparin (unfractionated or low molecular weight; LMWH) and nitrates (sub-lingual or intravenous). All patients are given a statin for secondary prevention and should their blood pressure allow, all are prescribed an angiotensin converting enzyme inhibitor (ACE inhibitor) or an angiotensin receptor blocker (ARB). This treatment is continued for 48 hours provided the patient remains pain free. Cardiac enzymes are taken at least once, six to 12 hours after the index pain.

Should the patient be haemodynamically unstable or experience on-going ischaemia (on-going/recurrent chest pain or dynamic ischaemic ECG changes), referral to a tertiary centre for angiography is indicated. Patients with a TIMI score^11^ of 5 or more are also referred.

Should the patient remain asymptomatic on medical management, heparin anticoagulation is discontinued after 48 hours and the patient is mobilised. If the patient develops recurrence of ischaemic chest pain on mobilisation, referral to a tertiary centre for angiography follows. Should the patient mobilise without complication, a sub-maximal exercise stress test (EST) is performed pre-discharge where possible to exclude poor prognostic features, which also dictate referral. Patients who do not demonstrate any of these features are referred to the tertiary centre as out-patients.

The current best-practice guidelines therefore aim to identify a small group of very high-risk patients who are referred for early angiography, whereas medical management is considered sufficient for those who stabilise on heparin anticoagulation and mobilise without complication. This is regardless of the troponin level, which is for prognostic purposes only; exposing a significant proportion of patients who would be classified as high risk according to the ESC guidelines to potentially sub-optimal care according to these guidelines.^1^

Despite these clear local best-practice guidelines, very little is known regarding the demographics, actual management and referral patterns of patients suffering an NSTEMI in South Africa and how this influences the outcome of those patients. This study aimed to determine whether the management of an NSTEMI differs depending on the hospital to which the patient presents (patients presenting to secondary hospitals being less likely to receive early invasive management), and if so, whether this is a consequence of geographical remoteness or level of care, and how this influences outcome.

## Methods

After obtaining ethical approval, including a waiver of informed consent from the University of Stellenbosch’s Health Research Ethics Committee (reference no: N11/09/288), a retrospective cohort study was conducted looking at adults presenting with NSTEMIs to TBH, KBH and WH. This was done over a one-year period from September 2010 to August 2011. Patients presenting during the first six months of the study time were analysed in terms of clinical risk profile and in-patient management, and then subsequent management up to six months post admission.

These hospitals were chosen for their unique similarities and differences. TBH is situated in Parow, Cape Town and is one of two academic referral centres in the city. It has 1 310 beds and provides a tertiary service to about 2.64 million people.^12^ In addition it provides a secondary service to the immediate surrounding areas, this latter group being the subject of this study. The Division of Cardiology within the Department of Medicine at TBH manages all ischaemic chest pain and has 28 beds with three full-time cardiologists.

KBH and WH are both secondary hospitals, similar except for their physical proximity to their tertiary referral centre, namely TBH. KBH and WH have 282 and 269 beds, respectively, with 84 and 55 of those beds being assigned to the Departments of Medicine. Both hospitals have two full-time specialist physicians. Like TBH, KBH is also situated in Parow, 4.6 km from TBH, while WH is situated in the Boland/Overberg region of the Western Cape, approximately 94 km (over an hour) away from TBH.

Patients 18 years and older presenting to the Departments of Medicine at KBH and WH, and to the secondary service provided by the Division of Cardiology, Department of Medicine at TBH with an NSTEMI from September 2010 until February 2011 were included in this retrospective study. NSTEMI was defined as angina-type chest pain in an unstable pattern, requiring hospitalisation and associated with elevated troponin levels (troponin I ≥ 1.0 μg/l; troponin T ≥ 0.1 ng/ml) and no signs of ST-segment elevation.^1^

Patients with the following were excluded: renal failure (creatinine > 200 μmol/l), patients who developed an NSTEMI during hospitalisation for a condition other than ACS, including surgery within two weeks, cerebrovascular accident (CVA), anaemia (haemoglobin < 9 g/l), septicaemia (fever and evidence of systemic infection), warfarin therapy, known high bleeding risk, life expectancy less than six months, patients referred from other secondary hospitals for tertiary care. Previously documented left bundle branch block (LBBB) without new changes were allowed.

Cases were identified from the records of the National Health Laboratory Service at KBH, WH and TBH. All recorded positive cardiac troponin levels from September 2010 to February 2011 were collected. After obtaining permission from the chief medical superintendent, the original medical records of all these cases were requested and screened and those identified as having suffered an NSTEMI without exclusion criteria were included in the study.

**Fig. 1.**
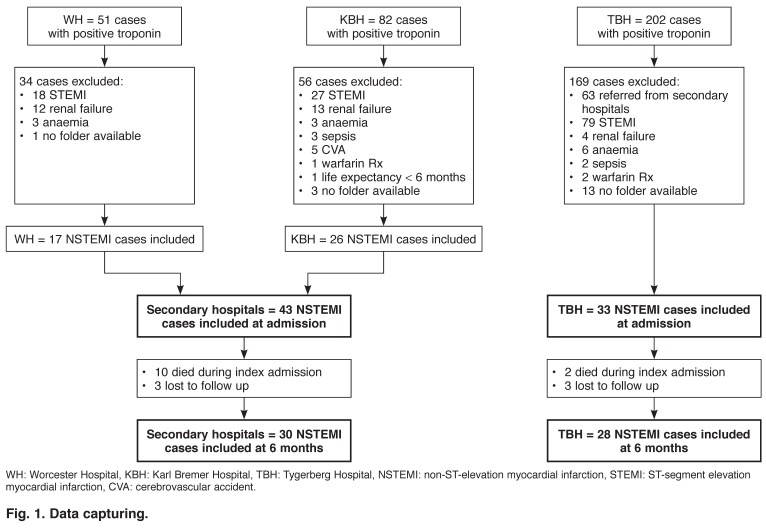
Data capturing.

Data were collected anonymously from the medical records of those cases identified for inclusion. Two data sets were collected, data during the index admission and data from follow-up visits over the following six months. Those cases for which no information was available at six months were included in the initial data set and documented as lost to follow up for the second data set (Fig. 1).

## Statistical analysis

The statistical analysis was done in conjunction with the University of Stellenbosch’s Centre for Statistical Consultation. Data from the two secondary-level hospitals (WH and KBH) were combined into a single data set, referred to as the secondary hospitals (SH). This data set was then analysed and compared with the TBH data. Descriptive statistics and chi-squared comparisons were done for categorical data. A *p*-value < 0.05 in a two-tailed test of proportions was considered significant.

Unless stated otherwise, continuous data is displayed as mean ± standard deviation (SD). Analysis of variance was done on this data and a *p*-value < 0.05 was considered significant. Where statistically significant differences in management or outcome were found between the SH cohort and the TBH cohort, a sub-analysis was done comparing WH and KBH to ascertain whether these differences were due to differences in management between these hospitals.

## Results

The baseline characteristics of the two groups were similar except for more documented dyslipidaemia and prior aspirin use in the TBH group (Table 1).

**Table 1:** Baseline Characteristics

	*Secondary hospitals n = 43 (%)*	*TBH n = 33 (%)*	p*-value*
Age (years) (± SD)	60.5 (± 12.6)	61.0 (± 14.88)	0.8774
Male	24 (56)	19 (58)	0.8779
Female	19 (44)	14 (42)	0.8779
Hypertension	33 (77)	23 (70)	0.4903
Diabetes mellitus	12 (28)	14 (42)	0.1868
Dyslipidaemia	16 (37)	20 (61)	0.0421*
Obesity	3 (7)	1 (3)	0.4319
Smoking	24 (56)	18 (52)	0.7993
Current	17 (40)	11 (33)	0.2641
Past	7 (16)	7 (21)	0.3873
COPD	3 (7)	2 (6)	0.8727
Ischaemic heart disease	16 (37)	17 (52)	0.2123
Stable angina pectoris	5 (12)	4 (12)	0.2123
Unstable angina pectoris	1 (2)	3 (9)	0.1905
Previous MI	10 (23)	9 (27)	0.6885
Previous angiography	9 (21)	10 (30)	0.3496
Previous stent	3 (7)	8 (18)	0.1340
Previous CABG	4 (9)	7 (21)	0.1436
Family history of IHD	4 (9)	5 (15)	0.4363
Prior asprin use within 7 days	20 (47)	23 (70)	0.0415*
Known stenosis > 50%	7 (16)	11 (33)	

COPD: chronic obstructive pulmonary disease, MI: myocardial infarction, CABG: coronary artery bypass graft. *p-values calculated comparing TBH and secondary hospital groups, *p* < 0.05 was statistically significant.

The groups were similar in terms of their clinical presentation (Table 2). In 58% of patients it was their first presentation with chest pain. There was a large variation in time to presentation from the onset of pain (mean: 24.21 ± 33.75 hours, median: 7 hours). On presentation, patients had a heart rate of 85.73 ± 24.85 beats per minute. Cardiac failure was documented in 39%, with a relatively equal distribution between Killip II, III and IV failure.^13^ Very few patients (5.26%) had a normal ECG, with the most frequent abnormality being ST-segment depression, seen in 46%.

**Table 2:** Clinical Presentation

	*Secondary hospitals n = 43 (%)*	*TBH n = 33 (%)*	p*-value*
Chest pain			
First episode	25 (58)	19 (58)	0.9606
Time to present (hours) (± SD)	19.65 (± 29.54)	30.15 (± 38.21)	0.1963
Duration of pain (mins) (± SD)	30.15 (± 38.21)	47.58 (± 35.51)	0.1488
Recurrence	23 (55)	14 (42)	0.2880
Pulse rate (beats per minute) (± SD)	84.16 (± 28.20)	87.84 (± 19.89)	0.4072
Systolic blood pressure (mmHg) (± SD)	130.67 (± 35.84)	144.84 (± 33.30)	0.0822
Diastolic blood pressure (mmHg) (± SD)	75.90 (± 19.81)	80.21 (± 17.91)	0.9568
ECG findings			
Normal	2 (5)	2 (6)	0.7859
Previous MI	9 (21)	9 (27)	0.5203
ST depression	19 (44)	16 (48)	0.7094
T-wave changes			
Flattening	6 (14%	1 (3)	0.0829
Inversion	10 (23)	11 (33)	0.3315
Dynamic changes	10 (23)	12 (36)	0.2129
Wellens’ syndrome	2 (5)	4 (12)	0.2322
Left ventricular hypertrophy	5 (12)	5 (15)	0.6536
Left bundle branch block (old)	4 (9)	4 (12)	0.6925
Right bundle branch block	6 (14)	4 (12)	0.8142
Atrial fibrillation	3 (7)	2 (6)	0.8727
Atrial flutter	0 (0)	1 (3)	0.2505
Finger-prick blood glucose (mmol/l)			
< 4 .0	1 (2)	0 (0)	0.3779
4.1–6.9	27 (63)	15 (45)	0.1319
7.0–10.0	8 (19)	6 (18)	0.9624
10.1–19.9	7 (16)	10 (30)	0.1459
≥ 20.0	0 (0)	2 (6)	0.1018
Temperature (°C)	35.85 (± 0.61)	36.10 (± 0.59)	0.0768
Cardiac failure	18 (42)	12 (36)	0.4740
Killip II	8 (19)	5 (15)	0.6919
Killip III	3 (7)	5 (15)	0.2497
Killip IV	7 (16)	2 (6)	0.1718
Serum creatinine (μmol/l) (± SD)	100.09 (± 35.46)	101.21 (± 33.75)	0.1938
TIMI score (± SD)	3.46 (± 1.42)	4.33 (± 1.08)	0.0046*
GRACE score (± SD)			
Probability of death			
in hospital	8.79 (± 14.60)%	5.49 (± 0.09)%	0.2321
at 6 months	15.57 (± 22.49)%	11.03 (± 12.68)%	0.2695
Probability of death or MI			
in hospital	18.81 (± 12.82)%	16.72 (± 8.94)%	0.4279
at 6 months	31.67 (± 19.59)%	28.39 (± 13.36)%	0.4119

*p-values calculated comparing TBH and secondary hospital groups, *p* < 0.05 was statistically significant.

Patients presenting to TBH had a significantly higher TIMI score than those presenting to the SH (*p* = 0.0046). This could not be accounted for by differences between WH and KBH, where the TIMI score was 3.412 ± 1.064 and 3.615 ± 1.134, respectively (*p* = 0.5587).^11^ This difference in risk stratification was not reflected in the Grace risk score.^14^

Most cases were treated with aspirin (87%) and LMWH (91%) (Table 3). Those presenting to TBH were more likely to receive early β-blockade than were those presenting to the SH (67 vs 35%, respectively, *p* = 0.0055). This could not be accounted for by a difference between WH and KBH where 41 and 31% of patients received β-blockers, respectively (*p* = 0.4839).

**Table 3:** Initial Medical Management

	*Secondary hospitals n = 43 (%)*	*TBH n = 33 (%)*	p*-value*
Aspirin	35 (81)	31 (94)	0.9558
150 mg	30 (70)	28 (85)	0.1253
300 mg	5 (12)	3 (9)	0.7209
β-blocker	15 (35)	22 (67)	0.0055*
Nitrates	25 (60)	17 (56)	0.6559
sub-lingual	16 (40)	9 (30)	0.4046
intravenous	9 (21)	8 (24)	0.7313
Heparin			
LMWH	37 (86)	32 (97)	0.0829
UFH	0 (0)	0	(0)
Morphine	7 (16)	4 (12)	0.6071
Dobutamine	6 (14)	1 (3)	0.0829

LMWH: low-molecular weight heparin, UFH: unfractionated heparin. *p-values calculated comparing TBH and secondary hospital groups, *p* < 0.05 was statistically significant.

While the clinical presentation and initial medical management was largely similar for the two cohorts, there were significantly more angiograms performed in the TBH group (94%) compared to the SH group (51%) (*p* < 0.0001) (Table 4). Again this was not due to differences in the frequency of invasive management between WH (48%) and KBH (54%) in sub-analysis (*p* = 0.6633). There was also no difference in the frequency of referral to TBH from WH or KBH (71 and 73%, respectively; *p* = 0.8588), and the acceptance rate of referrals was equally high from both hospitals (92% for WH and 94% for KBH, *p* = 0.7347). Cases from WH did however have a significantly longer time to angiography than those from KBH (3 ± 1.60 vs 1.5 ± 1.22 days respectively, *p* = 0.0225).

**Table 4:** Invasive Management On Index Admission

	*Secondary hospitals n = 43 (%)*	*TBH n = 33 (%)*	p*-value*
Angiography performed as in-patient	22 (51)	31 (94)	< 0.0001*
Time (days from admission) (±SD)	2.14 (± 1.52)	1.70 (± 1.65)	0.8615
Coronary revascularisation			
Via PCI (expressed as a % of angiography cases)	10 (45)	21 (68)	0.1018
Via PCI (expressed as a % of entire group)	10 (23)	21 (64)	0.0004*
Via PCI or CABG (% of angiography cases)	18 (82)	29 (94)	0.1842
Via PCI or CABG (expressed as a % of entire group)	18 (42)	29 (88)	<0.0001*
No. of stents	1.3 (± 0.48)	1.3 (± 0.91)	0.8952
Referred for CABG	8 (36)	8 (25)	0.0795

PCI: percutaneous coronary intervention, CABG: coronary artery bypass graft. *p-values calculated comparing TBH and secondary hospital groups, *p* < 0.05 was statistically significant.

At angiography, stenosis was seen in the left anterior descending artery (LAD) in 72% of cases, in the right coronary artery (RCA) in 72% and in the left circumflex (LCx) in 59%. In 80% of cases there was multi-vessel disease (40% double-vessel and 40% triple-vessel disease); 6% had small-vessel disease. If percutaneous intervention (PCI) was performed, the culprit lesion was the RCA in 21%, the LCx in 21% and the LAD in 17% of cases.

When angiography was performed, both cohorts were equally likely to receive coronary revascularisation via PCI (45% for the SH and 68% for TBH, *p* = 0.1018). The SH group had more stenosis of the left main stem (LMS) (*p* = 0.0477) and there was a trend for cases from the SH to be more frequently referred for coronary artery bypass grafting (CABG) (36 vs 26% in the TBH cohort, *p* = 0.0795), the majority as in-patients.

Patients presenting to TBH directly had a better in-hospital survival rate than those presenting to the SH (94 vs 77%, *p* = 0.0326) (Table 5). At six months there was a tendency to better survival in the TBH group (90 vs 73%, *p* = 0.0614). Most patients were discharged on aspirin, β-blockers, ACE inhibitors and statins and remained pain free; 23% of cases were re-admitted to hospital during follow up, most commonly with unstable angina pectoris (UAP) (54%, data not shown); 16% of cases underwent subsequent angiography.

**Table 5:** Outcomes At Discharge And At 6 Months

	*Secondary hospitals n = 43 (%)*	*TBH n = 33 (%)*	p*-value*
At Discharge			
Mortality	10 (23)	2 (6)	0.0326*
Discharge medications (% of survivors)			
Asprin	31 (94)	30 (97)	0.5918
β-blocker	29 (88)	30 (97)	0.1851
ACE inhibitor	25 (76)	26 (84)	0.4201
ARB	1 (3)	2 (6)	0.5175
Statin	30 (91)	29 (94)	0.6942
Spironolactone	3 (9)	2 (6)	0.6942
Clopidogrel	8 (24)	9 (29)	0.6646
Days in hospital (± SD) (% of survivors)	6.14 (± 4.33)	5.82 (± 5.26)	0.2100
At 6 months			
Survived (expressed as % of entire group)	29 (73)	27 (90)	0.0614
Survived (expressed as % of survivors at discharge)	29 (97)	27 (96)	0.9247
Chest pain (% of survivors at 6 months)			
None	17 (59)	16 (59)	0.6206
Occasional	9 (31)	10 (37)	0.8540
CCS 2	2 (7)	1 (4)	0.5960
CCS 3	1 (3)	0.(0)	0.3302
Readmission to hospital (% of survivors at 6 months)	9 (31)	4 (15)	0.1461
Subsequent angiography (% of survivors at 6 months)	6 (21)	3 (11)	0.3248
Time from admission (months)	3.86 (± 2.07)	1.33 (± 0.58)	0.0121*
Coronary revascularisation within 6 months (*n* = 43 and 33)	19 (44)	28 (85)	0.0001*

CCS: Canadian Cardiovascular Society angina classification *p-values calculated comparing TBH and secondary hospital groups, *p* < 0.05 was statistically significant.

As the numbers of patients re-admitted to hospital (nine for the SH group and four for TBH) and those undergoing angiography after discharge were small (six for the SH and three for the TBH cohort), it was not possible to perform a meaningful statistical analysis looking for differences between these groups; 66% of these cases received coronary revascularisation, either via PCI or subsequent CABG. Cases presenting to TBH underwent subsequent angiography after 1.33 ± 0.57 months, and those from the SH after 4.5 ± 2.07 months.

## Discussion

The management of patients suffering an NSTEMI presenting to the public health sector is affected by the level of service to which the patient presents. This is not a result of geographical remoteness from the tertiary centre or other differences in management between the secondary hospitals. Patients presenting to the secondary-level service provided by the Division of Cardiology at TBH were more likely to receive invasive in-patient management with coronary angiography than were those presenting to the SH (94 vs 51%, *p* < 0.0001). This difference was due to the fact that the secondary service at TBH is provided by the sub-specialist Division of Cardiology with immediate access to angiography. The difference in physical proximity to TBH between WH (94 km away) and KBH (4.6 km away) did not influence the accessibility of in-patient angiography, with cases being equally likely to be referred to and accepted by the Division of Cardiology at TBH from WH and KBH

The difference in the TIMI risk score for the two groups was a potential confounder in the analysis of why the TBH cohort received more angiography than the SH cohort, however both groups fell in the intermediate risk group, so the significance of this difference is unclear.^11^ The TIMI score was calculated by the investigators from the case records, as it was not uniformly documented. The difference in TIMI score between the TBH cohort and the SH cohort can be accounted for by the difference in frequency of documented dyslipidaemia and prior aspirin use in the two groups. This difference may be true, or it may be only an apparent difference due to the retrospective nature of the study.

As the baseline data were captured from patient records, only those cardiovascular risk factors documented could be captured. If there was no record of medications taken prior to admission, prior aspirin use could not be assumed. The fact that the difference between the two cohorts in the TIMI risk score^11^ was not reflected by the Grace risk score suggests that this might be a factor of documentation rather than one of clinical risk.^14^-^16^

This study also showed a difference in in-patient mortality between the two cohorts, with a higher mortality in the SH cohort. This was despite the fact that the TBH cohort had a greater risk for mortality within the first 14 days, as assessed by the TIMI risk score.^11^ As the only difference in management between the two groups was the initial use of β-blockers (although β-blocker use at discharge was similar in both groups), and in-patient invasive management, one must consider that one of these is responsible for the difference in mortality.

While β-blockers are well known to have benefit acutely in patients with ST-elevation myocardial infarctions (STEMIs),^17^,^18^ this is not known for NSTEMIs. On the other hand, it is well documented that early invasive management in patients suffering NSTEMIs improves survival.^2^-^6^ It is therefore reasonable to assume that the difference in access to in-patient coronary angiography between the TBH and SH groups was at least in part responsible for the difference in mortality between the two cohorts.

Comparing the study patients to the literature, both the TBH and SH cohorts had a higher mortality rate than expected, both in-hospital (6 and 23%, respectively) and at six months (10 and 27%, respectively). The literature predicts a mortality of 1.4–4.4% in hospital and 1.9–5.9% at six months to one year.^9^,^10^,^19^-^22^ Whether this was due to differences in in-patient angiography rates among our cases (51% for the SH group and 94% for TBH) compared to the literature remains unclear, as in-patient angiography was performed in 10–98% of patients in these trials.^10^,^19^-^22^

The predicted mortality rates for the two cohorts as calculated by the Grace risk score14 (in-patient mortality: 8.79 ± 14.60% and 5.49 ± 0.09%; mortality at six months: 15.57 ± 22.49% and 11.03 ± 12.68% for the SH group and TBH groups, respectively) was also higher than the mortalities expected from the literature, as quoted above.^9^,^10^,^19^-^22^ The patients in this study were a high-risk group as they all suffered an NSTEMI, whereas the trials quoted above looked at all non-ST elevation ACS (NSTE-ACS), and therefore included patients with UAP.

This study also demonstrated a high rate of cardiac failure. As the presence of cardiac failure was elicited from descriptions in the records, this may have been a true reflection of the study population or it may have been due to documentation. This may explain the high mortality rate in this study, both in reality and as predicted by the Grace risk score.^14^

When examining the two groups separately, the patients in the TBH cohort came closer to the mortality rate predicted by the Grace risk score,^14^ both in-hospital and at six months (6 vs 5% in-hospital and 10 vs 11% at six months, respectively) than did those in the SH cohort (23 vs 9% in-hospital and 28 vs 12% at six months, respectively), which would imply that the difference in management (either the increased frequency of angiography with in-hospital coronary revascularisation, or the earlier use of β-blockers, or both) was the cause of the difference in mortality, as previously discussed. Referring to earlier literature,^10^,^19^,^22^,^23^ the rates of angiography in the TBH (94%) and SH cohorts (51%) were similar to and less than the rates of angiography in the early invasive arms of these trials (96–98%),^10^,^19^,^22^,^23^ respectively. However, the conservative arms of these trials had lower rates of coronary angiography (11–51%) with a lower mortality rate.^10^,^19^,^22^,^23^ This implies additional factors contributing to the poorer survival in the South African state hospital setting.

The conservative arms in these earlier international trials^10^,^19^,^23^ included other anti-platelet agents in addition to aspirin (ADP receptor antagonists or glycoprotein IIb IIIa inhibitors), as recommended by the ESC.^1^ At the time of writing both of these agents were only available to patients undergoing angiography in the state hospital setting in the Western Cape. They are not available for medical management of an NSTE-ACS, even for those at high risk with NSTEMIs. Whether this was the cause of the higher-than-expected mortality rate in this study is not addressed, and further research into this question needs to be performed.

Examining specifically those cases in the SH cohort who died during the index admission, a 23.6 ± 21.98% probability of in-hospital mortality was predicted by the Grace score.^14^ This suggests that the recorded mortality rate was high in comparison with previous studies. Only half of these 10 cases were referred to TBH, and four of the five referred patients were accepted. It appears from this that lack of referral (50%) of those patients who subsequently died may in part be responsible for a poor outcome. This discrepancy in referral rate and acceptance rate is reflected in the cohort as a whole as well (72% referral rate and 94% acceptance rate). The lack of referral for tertiary care was likely to have been a contributing factor to the relative lack of in-patient angiography and coronary revascularisation in the SH group, and hence the higher in-patient mortality rate.

This study did not investigate the reasons for referral or lack thereof, as this information was difficult to obtain retrospectively. Further research into this should be done in order to fully address the high mortality rate in the SH cohort.

When looking at coronary revascularisation rates in the two groups, it would appear that the rates of PCI were low in both groups (45% for SH and 68% for TBH). This can be explained by the fact that a high percentage of patients (40%) had triple-vessel disease, and when coronary revascularisation is considered in total [via PCI or coronary artery bypass grafting (CABG)], the rates of revascularisation increased to 82 and 94% for the SH and TBH groups, respectively, suggesting that lack of coronary revascularisation was not a contributing factor in the high mortality.

## Limitations

This study had several significant limitations. The retrospective nature of the study design left the investigators dependent on clinical records for all data capturing. Record keeping is often less than optimal and significant information such as patients’ use of medications prior to admission was often not available in the records. This could render the baseline characteristics and clinical risk stratification of the patients unreliable, limiting the comparison between the two groups.

The sample size also limited the study findings. A number of factors contributed to a small sample size. The study intentionally targeted a high-risk group of patients with NSTEMIs. Due to the significant budget constraints experienced in the South African state healthcare system, many cases had only a single cardiac troponin value taken. Although it is part of the current best-practice guidelines that should only one troponin value be requested, it is taken six to 12 hours after the index event. There was no record in the clinical notes documenting that this was practiced. It was not possible to ascertain when the troponin samples were taken, and therefore cases may have been missed.

There were also a number of cases that were excluded due to the inability to obtain the patient’s folder (four for the SH cohort and 13 for the TBH cohort). No information was available for these cases and while it is not known whether these patients suffered an NSTEMI, it does raise concerns regarding the validity of the data. As the investigators were particularly interested in access to in-patient coronary angiography, there were strict exclusion criteria which also contributed to the small number of cases.

The small sample in the WH and KBH groups limited the data analysis in that the two groups had to be combined into a single cohort for analysis. Although sub-analysis was done comparing the WH and KBH groups when statistically significant differences were found and this did not reveal any differences between these two groups, the lack of differences may have been a factor of the sample size. The combination of WH and KBH into a single cohort may also have masked differences between these groups had they been independently compared to the TBH cohort.

## Conclusion

Despite these limitations, the study did reveal some significant differences in the in-patient management of patients presenting to secondary services at a tertiary centre (TBH) compared to patients presenting to secondary-level centres (KBH and WH). These included less initial β-blocker use, less in-patient invasive management and a higher in-patient mortality rate in the SH group. The lack of difference between the KBH and WH groups in this regard suggests that geographical proximity to (or remoteness from) the tertiary centre (TBH) was not a significant factor determining access to coronary angiography. Clearly factors other than geography and distance, specific to the level of service, were influencing both access to in-patient coronary angiography and in-patient mortality rates.

While it is well established that early angiography has a clear mortality benefit in patients suffering a NSTEMI,^2^-^6^ and current research is investigating the optimal timing for angiography; access to in-patient angiography remains problematic in the state hospital setting in the Western Cape, South Africa. The relative lack of access to coronary angiography for patients presenting to secondary-level hospitals, regardless of their geographical proximity to the tertiary centre TBH, results in an adverse mortality outcome for these patients. Coronary angiography remains a scarce resource, with three cardiologists in the Division of Cardiology, Department of Medicine at TBH providing a tertiary service to a population of 2.64 million.^12^ This inequality in access to in-patient invasive management needs to be addressed as a priority.
